# Whole-Blood Gene Expression Profile After Hypoxic-Ischemic Encephalopathy

**DOI:** 10.1001/jamanetworkopen.2023.54433

**Published:** 2024-02-02

**Authors:** Paolo Montaldo, Constance Burgod, Jethro A. Herberg, Myrsini Kaforou, Aubrey J. Cunnington, Asuncion Mejias, Grazia Cirillo, Emanuele Miraglia Del Giudice, Carlo Capristo, Prathik Bandiya, Chinnathambi N. Kamalaratnam, Rema Chandramohan, Swati Manerkar, Ranmali Rodrigo, Samanmali Sumanasena, Vaisakh Krishnan, Stuti Pant, Seetha Shankaran, Sudhin Thayyil

**Affiliations:** 1 Centre for Perinatal Neuroscience, Department of Brain Sciences, Imperial College London, London, United Kingdom; 2 Department of Women's and Children's Health and General and Specialized Surgery, University of Campania Luigi Vanvitelli, Naples, Italy; 3 Section of Paediatric Infectious Disease and Centre for Paediatrics and Child Health, Department of Infectious Disease, Imperial College London, London, United Kingdom; 4 Department of Infectious Diseases, St Jude Children’s Research Hospital, Memphis, Tennessee; 5 Center for Vaccines and Immunity, Abigail Wexner Research Institute at Nationwide Children’s Hospital, Columbus, Ohio; 6 Department of Neonatology, Indira Gandhi Institute of Child Health, Bengaluru, India; 7 Institute of Child Health, Department of Neonatology, Madras Medical College, Chennai, India; 8 Department of Neonatology, Lokmanya Tilak Municipal Medical College, Mumbai, India; 9 Department of Pediatrics, University of Kelaniya, Colombo, Sri Lanka; 10 Neonatal-Perinatal Medicine, Wayne State University, Detroit, Michigan

## Abstract

**Importance:**

Induced hypothermia, the standard treatment for hypoxic-ischemic encephalopathy (HIE) in high-income countries (HICs), is less effective in the low-income populations in South Asia, who have the highest disease burden.

**Objective:**

To investigate the differences in blood genome expression profiles of neonates with HIE from an HIC vs neonates with HIE from South Asia.

**Design, Setting, and Participants:**

This case-control study analyzed data from (1) a prospective observational study involving neonates with moderate or severe HIE who underwent whole-body hypothermia between January 2017 and June 2019 and age-matched term healthy controls in Italy and (2) a randomized clinical trial involving neonates with moderate or severe HIE in India, Sri Lanka, and Bangladesh recruited between August 2015 and February 2019. Data were analyzed between October 2020 and August 2023.

**Exposure:**

Whole-blood RNA that underwent next-generation sequencing.

**Main Outcome and Measures:**

The primary outcomes were whole-blood genome expression profile at birth associated with adverse outcome (death or disability at 18 months) after HIE in the HIC and South Asia cohorts and changes in whole-genome expression profile during the first 72 hours after birth in neonates with HIE and healthy controls from the HIC cohort. Blood samples for RNA extraction were collected before whole-body hypothermia at 4 time points (6, 24, 48, and 72 hours after birth) for the HIC cohort. Only 1 blood sample was drawn within 6 hours after birth for the South Asia cohort.

**Results:**

The HIC cohort was composed of 35 neonates (21 females [60.0%]) with a median (IQR) birth weight of 3.3 (3.0-3.6) kg and gestational age of 40.0 (39.0-40.6) weeks. The South Asia cohort consisted of 99 neonates (57 males [57.6%]) with a median (IQR) birth weight of 2.9 (2.7-3.3) kg and gestational age of 39.0 (38.0-40.0) weeks. Healthy controls included 14 neonates (9 females [64.3%]) with a median (IQR) birth weight of 3.4 (3.2-3.7) kg and gestational age of 39.2 (38.9-40.4) weeks. A total of 1793 significant genes in the HIC cohort and 99 significant genes in the South Asia cohort were associated with adverse outcome (false discovery rate <0.05). Only 11 of these genes were in common, and all had opposite direction in fold change. The most significant pathways associated with adverse outcome were downregulation of eukaryotic translation initiation factor 2 signaling in the HIC cohort (*z* score = −4.56; *P* < .001) and aldosterone signaling in epithelial cells in the South Asia cohort (*z* score = null; *P* < .001). The genome expression profile of neonates with HIE (n = 35) at birth, 24 hours, 48 hours, and 72 hours remained significantly different from that of age-matched healthy controls in the HIC cohort (n = 14).

**Conclusions and Relevance:**

This case-control study found that disease mechanisms underlying HIE were primarily associated with acute hypoxia in the HIC cohort and nonacute hypoxia in the South Asia cohort. This finding might explain the lack of hypothermic neuroprotection.

## Key Points

**Question:**

Do genome expression profiles at birth in neonates with hypoxic-ischemic encephalopathy (HIE) in a high-income country (HIC) differ from those of their counterparts in South Asia, and can this difference explain the lack of hypothermic neuroprotection?

**Findings:**

In this case-control study of 134 neonates with HIE from an HIC or South Asia and 14 healthy controls from the HIC, neonates in the HIC cohort who had an adverse outcome after HIE had a different host genome expression profile at birth compared with neonates in the South Asia cohort and displayed opposite regulation of the significant genes in common.

**Meaning:**

Findings of this study suggest that differences in the nature and timing of cerebral hypoxia ischemia explain the lack of hypothermic neuroprotection in South Asia.

## Teaser

This case-control study compares the genome expression at birth and associated outcome in neonates with hypoxic-ischemic encephalopathy in a high-income country (Italy) and their counterparts in low- to middle-income countries (India, Sri Lanka, and Bangladesh).

## Introduction

Hypoxic-ischemic encephalopathy (HIE) is a leading cause of death and disability among neonates born at full term. Worldwide, approximately 1 million neonates die or survive with a major disability every year from HIE.^1^ South Asia, particularly India, shoulders the highest disease burden, accounting for 60% of all HIE-related deaths in the world.^2,3^ Between 2010 and 2019, although neonatal mortality decreased by 32% in India, HIE-specific mortality remained unchanged, resulting in an economic loss of US $60 to $135 billion.^3^

Whole-body hypothermia has been associated with reduced death or disability after moderate or severe HIE^4^ in high-income countries (HICs) and is the standard treatment. However, the Hypothermia for Encephalopathy in Low-Income and Middle-Income Countries (HELIX) trial,^5^ the largest clinical trial of hypothermia in the world that was conducted in South Asia, reported that hypothermia did not reduce brain injury but increased mortality. Whole-body hypothermia was administered using servocontrolled devices in the HELIX trial, and attainment of the target temperature was earlier than in hypothermia trials in HICs.^6^ Nonetheless, hypothermia was not neuroprotective, regardless of the duration of birth depression or birth acidosis^7^ and place of birth.^6^ It is not known whether the differential response to whole-body hypothermia between HICs and South Asia is associated with the underlying disease mechanisms. Whole-blood gene expression profile is increasingly used to characterize host responses in various infectious diseases,^8,9,10,11^ including neonatal cytomegalovirus infections,^12^ and in HIE wherein the gene profiles are different for healthy neonates vs those with sepsis.^13,14^

In this study, we investigated the differences in blood genome expression profiles of neonates with HIE from an HIC vs neonates with HIE from South Asia, to understand why one population may benefit from hypothermic neuroprotection and another may not. In addition, we assessed the temporal changes in genome expression profile over the first 72 hours after birth in neonates with HIE compared with matched healthy controls in an HIC.

## Methods

### Study Design

This case-control study analyzed data collected from (1) a prospective observational study involving consecutive neonates with moderate or severe HIE who underwent whole-body hypothermia at the University Hospital of Campania in Italy between January 2017 and June 2019 (hereafter, the HIC cohort) and contemporary age-matched term healthy controls and (2) the HELIX randomized clinical trial, which recruited 408 neonates with moderate or severe HIE from 7 tertiary public sector neonatal intensive care units in India, Sri Lanka, and Bangladesh between August 2015 and February 2019 (hereafter, the South Asia cohort).^5^ The Imperial College London Research Ethics Committee and the local research ethics committees at all sites approved both previous studies and the present case-control study. Informed consent was obtained from parents prior to recruitment. We followed the Strengthening the Reporting of Observational Studies in Epidemiology (STROBE) reporting guideline.

In the HIC cohort, blood samples for RNA extraction were collected before initiation of whole-body hypothermia (within 6 hours after birth) and then again after 24, 48, and 72 hours. Age-matched term healthy controls were recruited from postnatal wards and had normal neonatal examination (eMethods in Supplement 1). In the South Asia cohort, there was only 1 blood sample drawn within 6 hours after birth, before initiation of hypothermia. Healthy controls could not be recruited in South Asia.

### Sample Collection and Processing

Peripheral venous or arterial blood samples were collected using customized blood RNA tubes (PAXgene; PreAnalytiX),^14^ frozen, and then later extracted. The RNA from samples underwent next-generation sequencing using sequencers (Illumina). Details of the sequencing method, quality control, and analysis are provided in the eMethods, eTable 1, and eFigures 1-3 in Supplement 1.

### Clinical Assessments

Within 6 hours after birth, all neonates with HIE were examined by certified examiners using the Eunice Kennedy Shriver National Institute of Child Health and Human Development Neonatal Research Network hypothermia trial encephalopathy staging (modified Sarnat staging),^15^ and the stage of encephalopathy was classified. Detailed neurodevelopmental examination between 18 to 24 months of age using the Bayley Scales of Infant Development III was performed by a certified examiner. Adverse outcome was defined as death or moderate or severe disability^15^ (eMethods in Supplement 1).

### Statistical Analysis

We used R, version 4.1.0 (R Project for Statistical Computing), for data analysis. Differential expression analysis to identify the genome expression profile of neonates with adverse neurological outcome was carried out with DESeq2, version 1.32.0 (Bioconductor) first unadjusted and then adjusted for birth weight, sex, and gestational age and treatment received (whole-body hypothermia vs usual care in the South Asia cohort). The R package maSigPro was used to identify genes that were differentially expressed over time, which were then clustered according to their pattern of expression. We corrected all of the *P* values for multiple testing with the Benjamini-Hochberg false discovery rate (FDR) method to control for type I error (5% FDR). Pathway analyses were conducted by using the differentially expressed genes (DEG; FDR <0.05 and absolute log_2_ fold change >1), which were associated with biological functions in the Ingenuity Pathways Knowledge Base (Ingenuity Systems)^16^ (eMethods in Supplement 1).

Statistical analyses for clinical variables were performed with SPSS Statistics, version 24 (IBM and SPSS Inc). Differences in continuous variables between groups were assessed with Mann-Whitney or unpaired, 2-tailed *t* test, as appropriate. Two-sided *P* < .01 was considered to be statistically significant for clinical variables. Data were analyzed between October 2020 and August 2023.

## Results

### Patient Characteristics

The HIC cohort was composed of 35 neonates (21 females [60.0%], 14 males [40.0%]) with a median (IQR) birth weight of 3.3 (3.0-3.6) kg and gestational age of 40.0 (39.0-40.6) weeks. The South Asia cohort consisted of 99 neonates (42 females [42.4%], 57 males [57.6%]) with a median (IQR) birth weight of 2.9 (2.7-3.3) kg and gestational age of 39.0 (38.0-40.0) weeks. Healthy controls included 14 neonates (9 females [64.3%], 5 males [35.7%]) with a median (IQR) birth weight of 3.4 (3.2-3.7) kg and gestational age of 39.2 (38.9-40.4) weeks (Table; eTable 2 in Supplement 1).

**Table.** Clinical Characteristics of Neonates With Hypoxic-Ischemic Encephalopathy (HIE) From a High-Income Country (HIC) or South Asia

Characteristic	Neonates with HIE, No. (%)		*P* value (HIC vs South Asia)^a^	Healthy controls in HIC cohort, No. (%) (n = 14)	*P* value (with HIE vs healthy controls)^a^
	HIC cohort (n = 35)	South Asia cohort (n = 99)			
Maternal					
Hypertension	3 (8.6)	7 (7.0)	.40	0	.02
Diabetes	1 (2.8)	2 (2.0)	.48	0	.04
Hypothyroidism	2 (5.7)	1 (1.0)	.16	0	.05
Intrapartum sentinel events	12 (34.3)	16 (16.2)	.02	0	.29
Neonatal					
Emergency cesarean delivery	16 (45.7)	27 (27.3)	.05	0	.002
Sex					
Female	21 (60.0)	42 (42.4)	.08	9 (64.3)	>.99
Male	14 (40.0)	57 (57.6)	.08	5 (35.7)	>.99
Birth weight, median (IQR), kg	3.3 (3.0-3.6)	2.9 (2.7-3.3)	<.001	3.4 (3.2-3.7)	.52
Gestational age, median (IQR), wk	40.0 (39.0-40.6)	39.0 (38.0-40.0)	.008	39.2 (38.9-40.4)	.92
Umbilical cord pH, median (IQR)	7.0 (6.9-7.0)	7.1 (6.8-7.3)	<.001	NA	NA
5-min Apgar score, median (IQR)	6.5 (5.0-8.0)	5.0 (4.0-6.0)	<.001	10.0 (9.5-10.0)	<.001
Intubation at birth	14 (40.0)	50 (50.5)	.32	0	.01
Clinical course					
Moderate encephalopathy	31 (88.6)	76 (76.8)	.15	0	NA
Severe encephalopathy	4 (11.4)	23 (23.2)	.15	0	NA
Induced hypothermia (sample collection within 6 h after birth)	35 (100.0)	44 (44.4)	NA	0	NA
Seizures^b^	10 (28.6)	87 (87.9)	<.001	0	NA
Age of onset of seizures, median (IQR), h	18.0 (13.2-25.3)	3.0 (1.0-5.0)	.002	NA	NA
Early-onset culture-positive sepsis	0	3 (3.0)	.56	0	NA
MRI					
MRI available	32 (91.4)	62 (62.6)	<.001	0	NA
Basal ganglia or thalamic injury	8 (25.0)	16 (25.8)	>.99	NA	NA
White-matter injury	10 (31.3)	44 (71.0)	<.001	NA	NA
Cortical injury	4 (12.5)	15 (24.2)	.27	NA	NA
Outcomes at 18-22 mo					
Death	4 (11.4)	40 (40.4)	.002	NA	NA
Moderate or severe disability	7 (20.0)	11 (11.1)	.24	NA	NA
Death and moderate or severe disability	11 (31.4)	51 (51.5)	.04	NA	NA

Abbreviations: MRI, magnetic resonance imaging; NA, not applicable.

^a^ *P* < .01 was statistically significant.

^b^ Seizures were electrographic and/or clinical in an HIC, whereas seizures were only clinical and electroencephalographic data were not obtained in South Asia.

Pregnancy-related illnesses were similar in mothers of neonates with HIE in the HIC and South Asia cohorts. The median (IQR) umbilical cord pH (7.0 [6.9-7.0] vs 7.1 [6.8-7.3]; *P* < .001) was lower and the 5-minute Apgar score (6.5 [5.0-8.0] vs 5.0 [4.0-6.0]; *P* < .001) was higher in the HIC vs the South Asia cohort. The median (IQR) onset of seizures was earlier (3.0 [1.0-5.0] hours vs 18.0 [13.2-25.3] hours; *P* = .002) and more neonates had seizures within 6 hours after birth (87 of 99 [87.9%] vs 10 of 35 [28.6%]; *P* < .001) in the South Asia cohort compared with the HIC cohort. The neonates from South Asia had more white-matter injury on magnetic resonance imaging (44 of 62 [71.0%] vs 10 of 32 [31.3%]; *P* < .001) and higher mortality (40 of 99 [40.4%] vs 4 of 35 [11.4%]; *P* = .002) at 18 months. Healthy controls did not have any maternal morbidities or intrapartum sentinel events.

### Comparison of Genome Expression Profile at Birth Associated With Adverse Outcome

This analysis included 35 neonates with HIE (28 with adequate quality samples taken within 6 hours after birth) from the HIC cohort and 99 from the South Asia cohort. Although the neonates were normothermic at the blood sample draw within 6 hours after birth, all 35 neonates in the HIC cohort and 44 (44.4%) in the South Asia cohort subsequently received whole-body hypothermia. The clinical characteristics of South Asia neonates with (n = 99) vs without (n = 309) genome expression data were not different (eTable 3 in Supplement 1).

**Figure 1.**
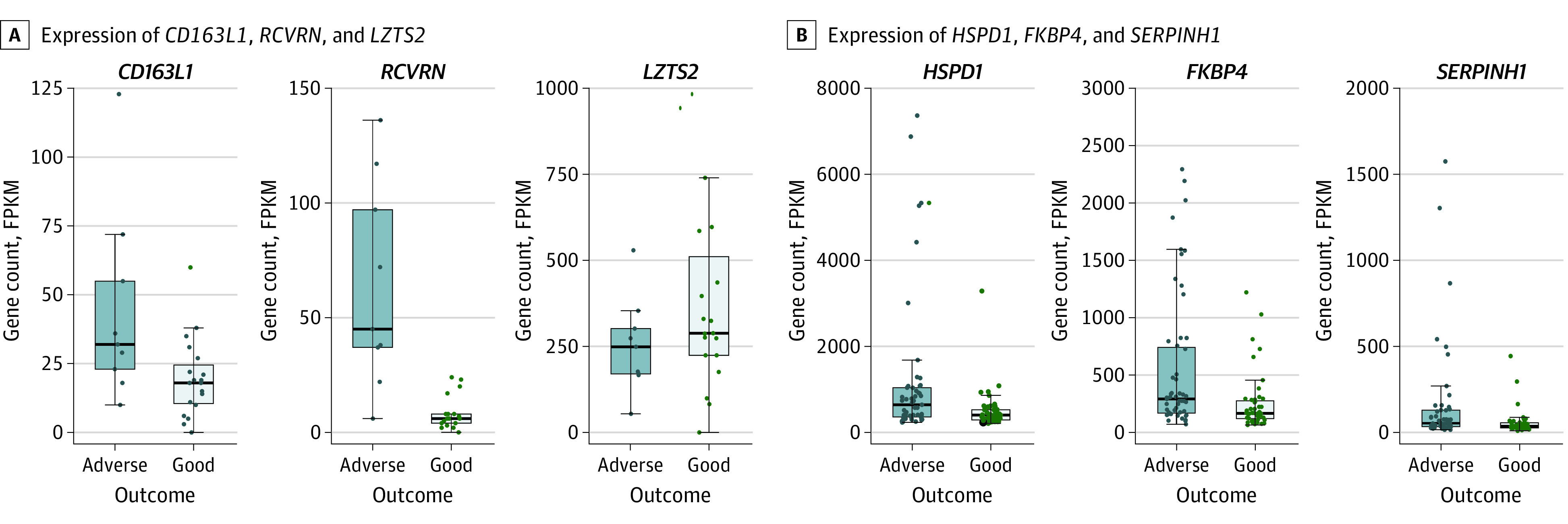
Comparison of Neonates With Hypoxic-Ischemic Encephalopathy With Adverse and Good Outcomes Gene count values are expressed as fragments per kilobase of transcript per million mapped reads (FPKM). Each box plot constitutes upper and lower bounds representing the first and third quartiles and a horizontal line representing the median. Boundaries of the whiskers are based on the 1.5 IQR value; all data points outside the boundary are outliers. Each dot represents a patient’s gene count. A, *CD163L1, RCVRN,* and *LZTS2* were the 3 most significant genes after comparison of neonates with HIE with adverse and good outcomes in a high-income country (HIC). B, *HSPD1, FKBP4,* and *SERPINH1* were the 3 most significant genes after comparison of neonates with HIE with adverse and good outcomes in South Asia. All 3 genes displayed a higher expression in the adverse vs the good outcome group.

The genome expression data of the neonates who developed adverse outcome (death or disability at 18 months) (51 in South Asia cohort; 9 in HIC cohort) were compared with the genome expression data of the neonates with good outcome (48 in South Asia cohort; 19 in HIC cohort). A total of 2143 genes in the HIC cohort and 83 genes in the HIC cohort were differentially expressed (FDR <0.05) between adverse outcome and good outcome groups (eFigure 4 in Supplement 1; eTables 4 and 5 in Supplement 2). The top DEGs in the HIC cohort were *CD163L1* (FDR <0.001; log_2_ fold change, 1.89), *RCVRN* (FDR <0.001; log_2_ fold change, 2.84), and *LZTS2* (FDR = 0.001; log_2_ fold change, −0.96). In the South Asia cohort, the top DEGs were *HSPD1* (FDR <0.001; log_2_ fold change, 1.17), *FKBP4* (FDR = 0.002; log_2_ fold change, 1.18), and SE*RPINH1* (FDR = 0.002; log_2_ fold change, 1.36) (Figure 1). The most significant pathways associated with adverse outcome were eukaryotic translation initiation factor 2 signaling in the HIC cohort (*P* < .001; *z* score = −4.56) and aldosterone signaling in epithelial cells in the South Asia cohort (*P* < .001; *z* score = null). A list of the significant pathways is provided in eTables 6 and 7 in Supplement 2.

**Figure 2.**
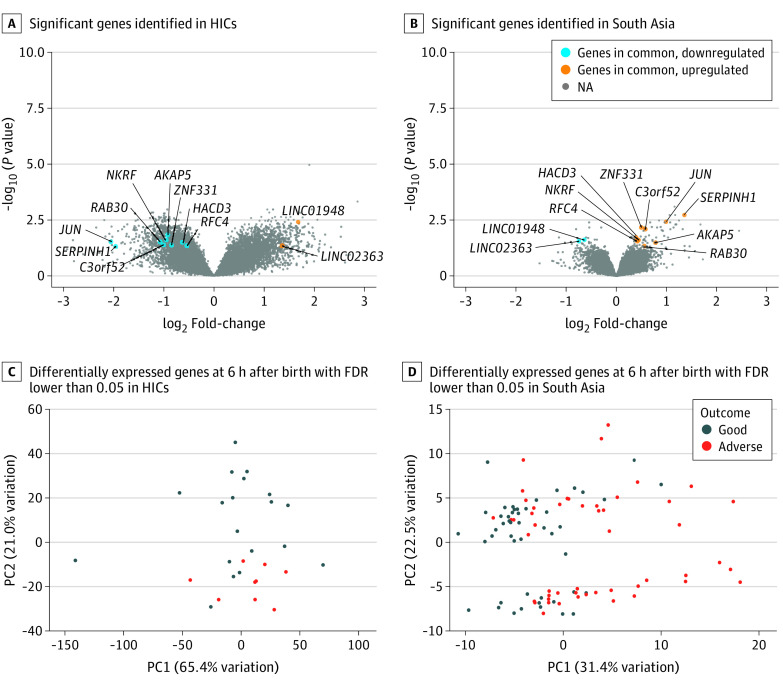
Genome Expression Profile After Birth in Neonates With Hypoxic-Ischemic Encephalopathy (HIE) With Adverse Outcomes FDR indicates false discovery rate; HIC, high-income country; PC1, first principal component; PC2, second principal component.

We then repeated the analysis by using only the genes with nonzero counts and that were in common between the South Asia and HIC cohorts (n = 30 121). A total of 1793 genes in the HIC cohort and 99 genes in the South Asia cohort were significantly differentially expressed (FDR <0.05). Only 11 significant genes were in common (eTables 8-10 in Supplement 2). None of the 11 genes had concordance in the direction of expression in both datasets compared with good outcome. *JUN*, SE*RPINH1*, *RAB30*, *C3orf52*, *AKAP5*, *NKRF*, *ZNF331*, *HACD3*, and *RFC4* were downregulated in the neonates from the HIC but upregulated in the neonates from South Asia. *LINC01948* and *LINC02363* were upregulated in the HIC and downregulated in South Asia (Figure 2A and B). Principal component analysis plots based on the DEG (1793 in the HIC cohort; 99 in the South Asia cohort) displayed a separation of the neonates with HIE with adverse outcome from neonates with good outcome, although this separation was clearer in the HIC than in South Asia cohort (Figure 2C and D).

### Longitudinal Genome Expression of Neonates Over First 72 Hours

**Figure 3.**
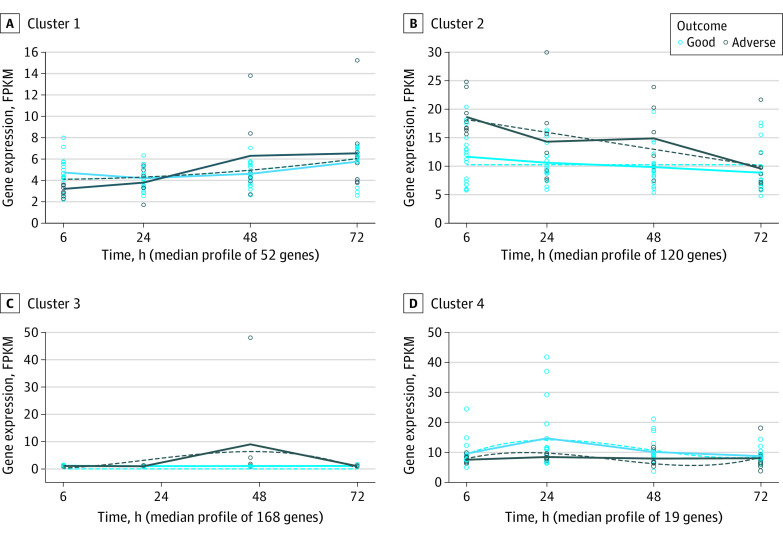
Temporal Evolution of Genome Expression of Neonates With Hypoxic-Ischemic Encephalopathy and Adverse Outcome Solid lines represent the actual mean values of gene expression at each time point. Fitted curves are shown as dashed lines. FPKM indicates fragments per kilobase of transcript per million mapped reads.

We evaluated how the genome expression profile changed over time in neonates with HIE in the HIC cohort who later developed adverse neurological outcomes and in neonates with HIE compared with healthy controls. A total of 104 samples collected at 4 different time points (6 hours, 24 hours, 48 hours, and 72 hours) from neonates with HIE in the HIC were included in this analysis. Differential expression identified 1604 significant DEGs between neonates with HIE with adverse outcomes vs their counterparts with good outcomes over time. A total of 359 genes fulfilled the *R*^2^ predefined cutoff value (eTable 11 in Supplement 2). These genes were clustered according to their similarities in expression patterns over time. A total of 4 clusters were identified (Figure 3). The most significant genes were SE*RPINE1* (FDR <0.001, cluster 3), *FN1* (FDR <0.001, cluster 3), and *COL4A1* (FDR <0.001, cluster 3).

In all 4 clusters, the difference between adverse and good outcome groups decreased after 72 hours with a similar expression profile (Figure 3). The top pathway of cluster 1 was enriched for cell cycle control of chromosomal replication. Cluster 2 was enriched in leukocyte extravasation signaling. Cluster 3 was enriched in pulmonary fibrosis idiopathic signaling pathway. Cluster 4 was enriched in cell cycle control of chromosomal replication. The list of significant pathways is provided in eTable 12 in Supplement 2.

**Figure 4.**
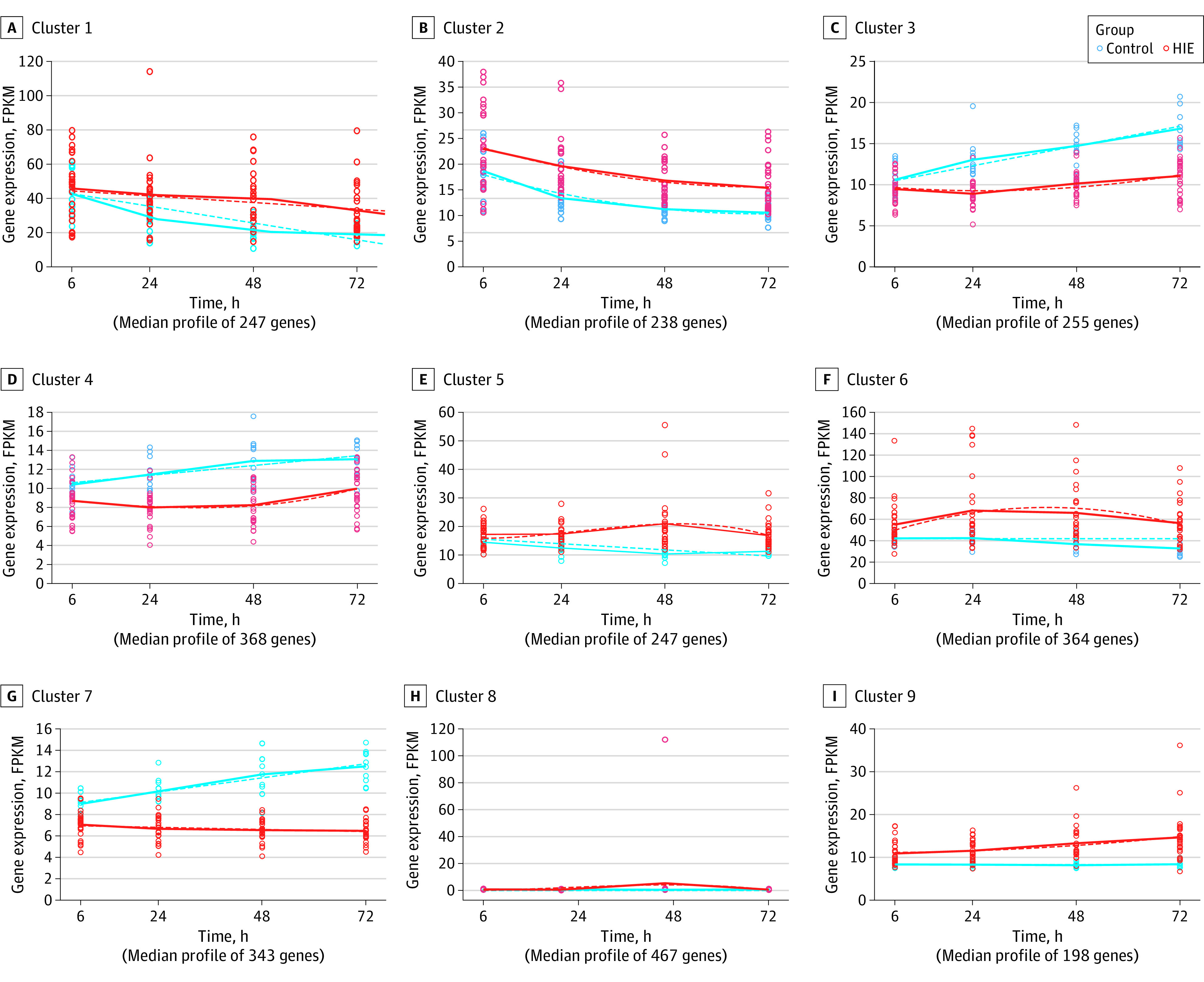
Temporal Evolution of Genome Expression of Neonates With Hypoxic-Ischemic Encephalopathy (HIE) vs Healthy Controls Solid lines represent the actual mean values of gene expression at each time point. Fitted curves are shown as dashed lines. FPKM indicates fragments per kilobase of transcript per million mapped reads.

A total of 150 samples (104 from neonates with HIE; 46 from healthy controls) collected at the 4 time points were included in this analysis. Differential expression over time identified 10 785 significant DEGs between neonates with HIE and controls. Significant genes were then clustered according to their similarities in expression pattern over time. A total of 2306 genes with the *R*^2^ predefined cutoff value were identified in 9 clusters (eTable 13 in Supplement 2; Figure 4; eFigure 5 in Supplement 1). The most significant genes were *CALHM6* (FDR <0.001, cluster 7), *TLR7* (FDR <0.001, cluster 7), and *PLEKHO1* (FDR <0.001, cluster 3) (eTable 13 in Supplement 2).

The top pathway of cluster 1 was enriched for p38 MAPK signaling (eTable 14 in Supplement 2). Cluster 2 was enriched for JAK family kinases in interleukin 6–type cytokine signaling. Cluster 3 was enriched for type 2 diabetes signaling. Cluster 4 was enriched for T_H_1 pathway. Cluster 5 was enriched for glycolysis I. Cluster 6 was enriched for ferroptosis signaling pathway. Cluster 7 was enriched for antigen presentation pathway. Cluster 8 was enriched for pulmonary fibrosis idiopathic signaling pathway. Cluster 9 was enriched for cell cycle control of chromosomal replication. See eTable 14 in Supplement 2 for these clusters.

## Discussion

We found that the whole-blood host genome expression profiles soon after birth associated with adverse outcomes in neonates with HIE from an HIC and neonates with HIE from South Asia were substantially different. Furthermore, the few common genes that were differentially expressed in both cohorts showed opposite associations with outcome. Downregulation of eIF2 at birth was associated with adverse outcome in the HIC cohort, whereas aldosterone signaling was associated with adverse outcome in the South Asia cohort. We also showed that neonates with HIE had a specific genome expression profile over the first 3 days after birth compared with healthy controls.

To our knowledge, this case-control study has been the largest study to explore genome expression profiles in HIE. The lack of concordance between the HIC and South Asia cohorts may suggest a different timing in the underlying mechanisms of intrapartum hypoxia ischemia between these neonates.

In the HIC cohort, the most significant genes associated with adverse outcomes within 6 hours after birth were *CD163L1, RCVRN*, and *LZTS2*. These genes are associated with the hypoxia-inducible factor-1α (HIF-1α) signaling, the master switch responsible for orchestrating the cellular response to acute hypoxia.^17,18^ *CD163L1* is a marker of the anti-inflammatory phenotype of macrophages and is mediated by interleukin 10, which is induced by HIF-1α.^19^ *RCVRN* forms part of the photoreceptors of the retina^20^ associated with hypoxia-induced retinogenesis.^21^ *LZTS2* interacts with the Wnt/β-catenin pathway to inhibit its transcriptional activity. It is upregulated in multiple forms of cancer-induced hypoxia through inhibition of the β-catenin activity, which is a pathway known to cross talk with HIF-1α.^22^ These patterns suggest an acute hypoxia ischemia in the HIC cohort.

In contrast, the most significant genes associated with adverse outcomes in South Asia were *HSPD1, FKBP4*, and SE*RPINH1*. These genes are mainly associated with oxidative stress and intermittent hypoxia resembling reperfusion injury, production of reactive oxygen species, and inflammation.^23,24^ *HSPD1*, a heat shock factor, is upregulated within 8 hours after short-term chronic hypoxia.^25^ *HSPD1* is also increased in patients with temporal lobe epilepsy in response to seizures.^26^ *FKBPs* are chaperone molecules and promote protein folding. Abnormal expression of SE*RPINH1* is considered a prognostic marker for cancer, and it is associated with immunoregulators and immune infiltration.^27^

The biological function of the DEG was also different between neonates with HIE from an HIC and neonates with HIE from South Asia. In the HIC cohort, the eIF2 signaling pathway was initially downregulated. In preclinical models, eIF2 phosphorylation occurs rapidly after neonatal hypoxia ischemia,^28^ leading to the inhibition of transcription and translation and to repression of protein synthesis shortly after the hypoxic-ischemic insult^29^ (downregulation). This phosphorylation reverses after reoxygenation, resulting in the resumption of protein synthesis (upregulation).^29^ On the other hand, aldosterone signaling was the most significant pathway identified in the South Asia cohort. This pathway has been implicated in chronic intermittent hypoxia settings, such as in obstructive sleep apnea, a chronic condition leading to intermittent hypoxia and subsequent activation of the renin-angiotensin-aldosterone system.^30^ Thus, this gene expression pattern suggests a nonacute nature of intrapartum hypoxia ischemia in South Asia. These findings agree with the changes to the genome expression previously reported in HIE.^13,14^ In a small cohort of 12 neonates with HIE in an HIC, the main biological pathways involved soon after birth indicated an acute oxygen deprivation as shown by the olfactory receptor response.^13^ Similarly, when the genome expression profile was examined in 47 infants with encephalopathy from South Asia, overrepresentation of genes involved in neuroinflammation was found.^14^ Each of these phases reflect a different timing of the disease mechanism starting with the acute hypoxic-ischemic insult; followed by a decrease of energy phosphates, oxidative stress, and apoptosis; and finally persistent inflammation and gliosis.

Subtle differences in the clinical phenotype, such as lower birth weight, lower incidence of intrapartum sentinel events, lesser birth acidosis, early onset of seizures, and higher white-matter injury on magnetic resonance imaging in neonates in South Asia vs an HIC, are consistent with the differences found on host genome expression profile. These data provide a mechanistic explanation for why whole-body hypothermia, the standard treatment for HIE in an HIC, was not neuroprotective but increased mortality among low-income populations in South Asia with the highest disease burden.

These data provide biological confirmation of our hypothesis about the compromised fetus in the womb, which explains that there is a high occurrence of HIE among low-income populations in South Asia and a lack of hypothermic neuroproection.^31^ Thus, the fetus is already compromised in the womb and is unable to cope with the normal hypoxic process of labor, especially if it is medically augmented.^32^ This clinical scenario, therefore, represents a nonacute hypoxia, as shown by preclinical models of growth-restricted animals and intermittent umbilical cord occlusion, where the seizure onset is earlier.^33^

This study also showed that the genome expression trajectory in neonates with HIE from an HIC who later develop adverse long-term neurological outcomes is different from those with favorable outcomes. In particular, increasing severity of outcomes was associated with upregulation of immunological and hypoxia-related pathways. In addition, the most significant genes (SE*RPINE1, FN1*, and *COL4A1*) over time in neonates with adverse outcome are all HIF1 gene targets, and their expression is substantially increased after oxidative stress.^34^ These data highlight that oxidative stress cascade is crucial in the extent of brain injury in neonates with HIE in an HIC, as it amplifies the cellular damage.

These data have implications for both HICs and South Asia. In an HIC cohort, adverse outcomes were still seen in 30% of the neonates with HIE despite whole-body hypothermia,^35^ highlighting that a one-size-fits-all strategy is not effective in HIE and that there is room for improvement in patient stratification, treatment, and management (ie, personalized diagnosis or treatments). In contrast, whole-body hypothermia was not neuroprotective and was possibly harmful in South Asia, and these neonates required different neuroprotective approaches that did not involve whole-body hypothermia.^36^ Host gene expression profiles may reflect the underlying etiological mechanisms and offer a unique approach for disease stratification. In particular, the trajectory of genome expression profile in neonates with HIE may help a prompt identification of those neonates at higher risk of adverse outcomes later. The role of gene expression as a diagnostic and stratification tool has been demonstrated in a range of diseases that are otherwise difficult to diagnose.^8,9,11,37,38^

### Limitations

Although, to our knowledge, this study is the first-ever detailed examination of genome expression profiles using in-depth next-generation sequencing in well characterized HIE cohorts from different continents, there are some limitations. First, the South Asian and HIC blood samples were sequenced in different laboratories and with different sequencers due to regulatory issues that prohibited the transport of blood samples out of India. Therefore, we could not analyze the 2 datasets together; instead, we performed an indirect comparison of the differential expression analyses. However, the same protocol was followed for both blood sample collection and RNA extraction. Second, the healthy controls were only from the HIC, again due to logistical and ethical challenges of collecting blood samples from healthy controls in South Asia. The challenge of recruiting healthy control infants led to similar limitations in previous studies with consistent results.^12,39^ Third, half of the neonates in South Asia underwent whole-body hypothermia as part of the HELIX trial. However, all the blood samples were collected before whole-body hypothermia was initiated and the differential expression analysis was adjusted for treatment. Hence, induced hypothermia is unlikely to have altered the preintervention genome expression data, although there may be an association with the final clinical outcome and allocation of each patient to the adverse outcome analysis group. Fourth, the HELIX trial was conducted in 3 South Asian countries, and its results may not be generalizable to other low- and middle-income countries. Fifth, although we collected blood samples on all 408 neonates recruited to the HELIX trial, only 99 had adequate-quality RNA suitable for next-generation sequencing. Many RNA samples were of poor quality due to breakdowns and temperature fluctuations of the deep freezers in South Asia and challenges of blood sample transport in dry ice. Nevertheless, there were no systematic differences between the neonates with RNA sequencing and those without.

## Conclusions

In this case-control study, unique differences in whole-blood genome expression at birth were found between neonates with HIE in an HIC and neonates with HIE in South Asia. The underlying mechanisms were associated with acute hypoxia in the HIC cohort and nonacute hypoxia in the South Asia cohort, which may explain the lack of hypothermic neuroprotection in latter settings. Moreover, the genome expression profile of neonates with HIE over the first 3 days after birth remained significantly different from the genome expression profile of healthy controls in an HIC. Whole-blood genome expression profile may be useful for rapid disease stratification for personalized neuroprotection in HIE and for monitoring therapeutic response.

## Notes

Supplement 1.
eMethods.
**eTable 1.** Clinical Variables of HIE Infants in Low-and-Middle-Income Countries in Batches 1 and 2 **eTable 2.** Mean (SD) Age and Mean (SD) Body Temperature at Blood Sample Collection **eTable 3.** Clinical Characteristics of Babies From the HELIX Trial (LMIC) With Gene Expression Data and That Were Included in the Analysis, and Those Without Gene Expression Data **eFigure 1.** Batch Effect Correction **eFigure 2.** Identification of Any Sources of Variance in the High-Income Countries Dataset **eFigure 3. **Identification of Any Sources of Variance in the Low-and-Middle-Income Countries Dataset **eFigure 4.** Genome Expression Profile After Birth in Neonates With HIE With Adverse Outcome **eFigure 5.** Temporal Evolution of Genome Expression of Neonates With HIE as Opposed to Healthy Controls

Supplement 2. **eTable 4. **Differentially expressed genes associated with adverse outcome in neonates with HIE in LMIC (FDR < 0.05) **eTable 5. **Differentially expressed genes associated with adverse outcome in neonates with HIE in HIC (FDR < 0.05) **eTable 6.** Reported are the results of the Ingenuity Pathway Analysis for the differentially expressed genes associated with adverse outcome at T0 (HIC) – adjusted **eTable 7. **Reported are the results of the Ingenuity Pathway Analysis for the differentially expressed genes associated with adverse outcome at T0 (LMIC) – adjusted **eTable 8.** Differentially expressed genes associated with adverse outcome in neonates with HIE in HIC (FDR < 0.05) **eTable 9. **Differentially expressed genes associated with adverse outcome in neonates with HIE in LMIC (FDR < 0.05) **eTable 10.** Differentially expressed genes associated with adverse outcome and in common between LMIC and HIC in neonates with HIE (FDR < 0.05) **eTable 11. **Differentially expressed genes between HIE neonates with adverse versus good outcome over time **eTable 12. **Cluster 1. Reported are the results of the Ingenuity Pathway Analysis for cluster 1 identified over the time when comparing adverse and good outcome (HIC) **eTable 12.** Cluster 2. Reported are the results of the Ingenuity Pathway Analysis for cluster 2 identified over the time when comparing adverse and good outcome (HIC) **eTable 12.** Cluster 3. Reported are the results of the Ingenuity Pathway Analysis for cluster 3 identified over the time when comparing adverse and good outcome (HIC) **eTable 12.** Cluster 4. Reported are the results of the Ingenuity Pathway Analysis for cluster 4 identified over the time when comparing adverse and good outcome (HIC) **eTable 13. **Differentially expressed genes between HIE neonates versus controls over time **eTable 14. **Cluster 1. Reported are the results of the Ingenuity Pathway Analysis for cluster 1 identified over the time when comparing HIE neonates and healthy controls (HIC) **eTable 14. **Cluster 2. Reported are the results of the Ingenuity Pathway Analysis for cluster 2 identified over the time when comparing HIE neonates and healthy controls (HIC) **eTable 14. **Cluster 3. Reported are the results of the Ingenuity Pathway Analysis for cluster 3 identified over the time when comparing HIE neonates and healthy controls (HIC) **eTable 14. **Cluster 4. Reported are the results of the Ingenuity Pathway Analysis for cluster 4 identified over the time when comparing HIE neonates and healthy controls (HIC) **eTable 14. **Cluster 5. Reported are the results of the Ingenuity Pathway Analysis for cluster 5 identified over the time when comparing HIE neonates and healthy controls (HIC) **eTable 14. **Cluster 6. Reported are the results of the Ingenuity Pathway Analysis for cluster 6 identified over the time when comparing HIE neonates and healthy controls (HIC) **eTable 14. **Cluster 7. Reported are the results of the Ingenuity Pathway Analysis for cluster 7 identified over the time when comparing HIE neonates and healthy controls (HIC) **eTable 14. **Cluster 8. Reported are the results of the Ingenuity Pathway Analysis for cluster 8 identified over the time when comparing HIE neonates and healthy controls (HIC) **eTable 14. **Cluster 9. Reported are the results of the Ingenuity Pathway Analysis for cluster 9 identified over the time when comparing HIE neonates and healthy controls (HIC)

Supplement 3.
Data Sharing Statement
