# Supplementary material for: Whole-Blood Gene Expression Profile After Hypoxic-Ischemic Encephalopathy
Source: JAMA Netw Open. 2024 Feb 2;7(2):e2354433. doi: 10.1001/jamanetworkopen.2023.54433 (PMC10837749; doi:10.1001/jamanetworkopen.2023.54433)
Supplement: Supplement 1. — eMethods. eTable 1. Clinical Variables of HIE Infants in Low-and-Middle-Income Countries in Batches 1 and 2 eTable 2. Mean (SD) Age and Mean (SD) Body Temperature at Blood Sample Collection eTable 3. Clinical Characteristics of Babies From the HELIX Trial (LMIC) With Gene Expression Data and That Were Included in the Analysis, and Those Without Gene Expression Data eFigure 1. Batch Effect Correction eFigure 2. Identification of Any Sources of Variance in the High-Income Countries Dataset eFigure 3. Identification of Any Sources of Variance in the Low-and-Middle-Income Countries Dataset eFigure 4. Genome Expression Profile After Birth in Neonates With HIE With Adverse Outcome eFigure 5. Temporal Evolution of Genome Expression of Neonates With HIE as Opposed to Healthy Controls [file jamanetwopen-e2354433-s001.pdf]

Supplemental Online Content

Montaldo P, Burgod C, Herberg JA, et al. Whole-blood gene expression profile after hypoxic-ischemic encephalopathy. *JAMA Netw Open*. 2024;7(2):e2354433. doi:10.1001/jamanetworkopen.2023.54433

eMethods.

eTable 1. Clinical Variables of HIE Infants in Low-and-Middle-Income Countries in Batches 1 and 2

eTable 2. Mean (SD) Age and Mean (SD) Body Temperature at Blood Sample Collection

eTable 3. Clinical Characteristics of Babies From the HELIX Trial (LMIC) With Gene Expression Data and That Were Included in the Analysis, and Those Without Gene Expression Data

eFigure 1. Batch Effect Correction

eFigure 2. Identification of Any Sources of Variance in the High-Income Countries Dataset

eFigure 3. Identification of Any Sources of Variance in the Low-and-Middle-Income Countries Dataset

eFigure 4. Genome Expression Profile After Birth in Neonates With HIE With Adverse Outcome

eFigure 5. Temporal Evolution of Genome Expression of Neonates With HIE as Opposed to Healthy Controls

This supplemental material has been provided by the authors to give readers additional information about their work.

© 2024 Montaldo P et al. *JAMA Network Open.*

eMethods

Healthy controls

In order to and understand the direction of expression (up- or down-regulation) during the time after hypoxic-ischaemic encephalopathy, healthy neonates were recruited. Neonates with risk factors of sepsis, receiving antibiotics, or those who required neonatal unit admission from any cause were excluded. Additional bloods were collected during blood group typing and serum bilirubin checks, as per the local policy.

Outcome definition

Severe disability was defined as any of the following: a Bayley III cognitive score < 70; a Gross Motor Function Classification System (GMFCS) level of 3 to 5; blindness; or hearing loss (inability to understand commands despite Amplification). Moderate disability was defined as a Bayley III cognitive score of 70 to 84 and either a GMFCS level of 2, seizure disorder or a hearing deficit requiring amplification to understand commands.

RNA sample extraction and processing

Peripheral venous or arterial blood (0.5 mL) were added to 1.4 mL ribonucleic acid -stabilizing solution (PreAnalytiX, Qiagen/BD) as per the manufacturer's instructions. The blood samples were initially stored at -20° C within 12 hours of collection and then transported to the local laboratory at -80° C. In both studies, each RNA sample was quantified using Qubit and run on a Tapestation to assess sample integrity. Ribosomal and mitochondrial RNA were removed via ribodepletion. After fragmentation, the sample was converted to double stranded DNA and ligated to Illumina adapters. Tapestation was used to assess fragment distribution and presence of artefacts.

The RNA samples from the HIC cohort were sequenced (Next generation sequencing) on an Illumina HiSeq4000 to generate 50M reads/sample (Imperial BRC Genomics Facility, Imperial College London).

The RNA samples from the LMIC cohort were sequenced in two separate batches; 55 on a Hiseq150 PE (Genotypic Technology Facility, Bangalore, India) and 44 on an Illumina Hiseq2500 (Medgenome Labs Ltd, Bangalore, India), both with 30M reads/sample[12]. The RNA from 10 neonates were sequenced twice for internal quality check and consistency. The clinical features of the infants in batch 1 and 2 were similar (**etable 1**).

Analyses

Analyses were carried out separately on samples with good quality RNA by following the same protocol in each cohort. Data was analyzed using 'R' Language and Environment for Statistical Computing (R) (v4.1.0). Principal Component Analysis (PCA) was used as part of the quality control process. The LMIC samples were first merged and any batch effect was then adjusted for using Combat-seq (sva v3.40.0). After batch correction and normalisation, PCA confirmed that batch effects had been removed (**eFigure 1**). Any sources of variance based on gestational age, gender and birth weight were also assessed (**eFigure 2, 3**).

We estimated normalisation factors and negative binomial dispersions from the raw count data. With these estimates, we adjusted negative binomial generalised linear models for each gene by conducting likelihood tests. The differential expression analysis was performed by using DESeq2 (v1.32.0) first unadjusted and then adjusted for for birth weight, gender and gestational age. For the LMIC dataset, the DESeq2 model was adjusted also for treatment (hypothermia or usual care) as well as for birth weight, gender and gestational age. All the neonates in HIC with HIE underwent therapeutic hypothermia. Therefore, no adjustment for treatment was performed. The p-values obtained were corrected for multiple testing using the Benjamini-Hochberg false discovery rate (FDR) method. As we wanted to investigate whether there was any overlap in the differentially expressed genes

© 2024 Montaldo P et al. *JAMA Network Open.*

between the two datasets (LMIC and HIC), we repeated the differential expression analysis by using only the genes with non-zero counts and which were in common between LMIC and HIC.

MaSigPro was used to perform a two-step regression model to identify significant genes differentially expressed over the time between a) Neonates with HIE and with adverse outcome versus those with good outcome and b) Neonates with HIE versus healthy controls. We used 3 degrees of freedom, a 0.2 R-squared cut-off value and good outcome and healthy controls as the comparator group, respectively. The procedure firstly defines a general regression model for the data and selects significant genes by computing a regression fit for each gene. This function also computes the p-value associated to the F-Statistic of the model, which is used to select significant genes. By default maSigPro corrects this p-value for multiple comparisons by applying the linear step-up, false discovery rate procedure. Secondly, stepwise regression is applied as a variable selection strategy to study differences between experimental groups and to find statistically significant different profiles. This approach allows for independent observations (different samples at different time points) and for unbalanced designs and heterogeneous sampling times.

We used Ingenuity Pathway Analysis software (QIAGEN) to assess the biological pathways enriched in the differentially expressed genes which fulfilled the criteria of an FDR <.05 and absolute log2 fold change>1 (canonical pathway analysis). Fisher's exact test was used to assess the significance of the canonical pathways and they were then ranked based on their P-values. The IPA regulation z-score algorithm was used to predict the activation state for a given biological function (increase or decrease).

© 2024 Montaldo P et al. *JAMA Network Open.*

eTable 1. Clinical variables of HIE infants in LMICs batches 1 and 2

Characteristic	LMICs batch 1 (n=55), No. (%)		LMICs batch 2 (n=44), No. (%)		P value
**Treatment group**	**Hypothermia**	**Normothermia**	**Hypothermia**	**Normothermia**	
	24 (44)	31 (56)	20 (45)	24 (54)	1.00
**Time of sample collection, mean (SD), h**	2.44 (1.68)		2.38 (1.45)		0.93
**Admission temperature, mean (SD), °C**	35.6 (1.5)	36.3 (1.0)	35.4 (1.4)	36.0 (0.7)	0.28
**Clinical seizures**	50 (91)		37 (84)		0.36
**Bloodstream positive early onset sepsis**	1 (2)		2 (4)		0.25
**Sentinel events**	12 (22)		4 (9)		0.10
**Onset of clinical seizures, median (IQR), h**	2 (1-6)		3 (1-4)		0.98
**Moderate encephalopathy**	43 (78)		33 (75)		0.81
**Severe encephalopathy**	12 (22)		11 (25)		0.81
**Gender (Males)**	30 (55)		27 (61)		0.54
**MRI available**	40 (73)		22 (50)		
**Basal ganglia/thalamic injury**	13 (32)		3 (14)		0.05
**White matter injury**	28 (70)		16 (72)		0.12
**Cortical injury**	12 (30)		3 (14)		0.03
**Adverse outcomes at 18 months**	27 (49)		24 (54)		0.67

© 2024 Montaldo P et al. *JAMA Network Open.*

eTable 2. Mean (SD) age and mean (SD) body temperature at blood sample collection

	**HIE in LMIC (n=99)**	**HIE in HIC (n=35)[a]**				**Healthy control neonates in HIC (n=14)[b]**			
	T0	T0	T1	T2	T3	T0	T1	T2	T3
	Before Hypothermia	Before Hypothermia	During Hypothermia			Normothermia			
Age (hours)	2.41 (1.57)	2 (1.4)	26 (2.4)	51 (2.7)	75 (2.4)	2.1 (1.7)	27 (2.3)	51 (2.4)	72 (2.1)
Temperature (°C)	35.9 (1.21)	35.8 (0.7)	33.5 (0.11)	33.5 (0.10)	33.5 (0.12)	36.6 (0.19)	36.7 (0.15)	36.7 (0.21)	36.7 (0.14)
Blood sample collection details									
All 4 time points	0	14	14	14	14	6	6	6	6
3 time points only	0	9	6	7	8	5	5	4	4
2 time points only	0	4	4	3	3	2	1	1	0
1 time point only	99	1	0	0	3	0	0	0	0
Total	99	28	24	24	28	13	12	11	10

[a]Good quality ribonucleic acid samples were available at 2 h (T0) before the start of hypothermia and subsequently at 26 h (T1), 51 h (T2) and 75 h (T3) after birth from 28, 24, 24 and 28 neonates respectively.
[b]Good quality RNA samples were available at 2 h (T0), 27 h (T1), 51 h (T2) and 72 h (T3) after birth from 13, 12, 11 and 10 neonates.
Abbreviations: HIC, high income countries; LMIC, low and middle-income countries.

© 2024 Montaldo P et al. *JAMA Network Open.*

eTable 3. Clinical characteristics of babies from the HELIX trial (LMIC) with gene expression data and that were included in the analysis, and those without gene expression data[a]

**Characteristic**	**Babies with gene expression data (n=99)**	**Babies without gene expression data (n=309)**	**P value**
Maternal details			
Hypertension	7 (7)	8 (3)	0.06
Diabetes	2 (2)	0	0.05
Hypothyroidism	1 (1)	0	0.24
Intra-partum sentinel events	16 (16)	27 (9)	0.05
Birth details			
Emergency LSCS	27 (27)	53 (17)	0.06
Males	57 (58)	210 (68)	0.06
Birth weight, kg	2.9 (0.4)	2.9 (0.4)	0.29
Gestational age, weeks	38.6 (1.4)	38.7 (1.3)	0.60
Cord pH	7.1 (6.8-7.3)	6.9 (6.8-7.0)	0.08
5 minutes Apgar	5.0 (4.0-6.0)	5.0 (4.0-5.0)	0.83
Intubation at birth	50 (50)	128 (41)	0.13
Clinical course			
Moderate encephalopathy	76 (77)	252 (82)	0.31
Severe encephalopathy	23 (23)	57 (18)	0.31
Induced hypothermia	44 (44)	158 (51)	0.25
Seizures[b]	87 (88)	275 (89)	0.71
Age of onset of seizures, h	3.0 (1.0-5.0)	2.0 (1.0-4.0)	0.31
Early onset sepsis	3 (3)	19 (6)	0.44
Magnetic resonance imaging			
MRI available	62 (63)	205 (66)	0.54
Basal ganglia/thalamic injury	16 (26)	47 (23)	0.71
White matter injury	44 (71)	168 (82)	0.15
Cortical injury	15 (24)	62 (30)	0.51
Outcome at 18 months			
Death	40 (40)	107 (35)	0.38
Moderate or severe disability	11 (11)	34 (11)	1.00
Death, moderate or severe disability	51 (51)	141 (46)	0.35

[a] Data are presented as number (percentage) unless otherwise indicated.
[b]Clinical seizures only.

© 2024 Montaldo P et al. *JAMA Network Open.*

eFigure 1. Batch effect correction

Principal component analysis of HELIX batch 1 (red) and HELIX batch 2 (green) after quantile normalization and ComBat-Seq (LMIC). Each dot represents a patient and is colored according to batch number. Square shapes represent good and triangle shapes represent adverse outcome.

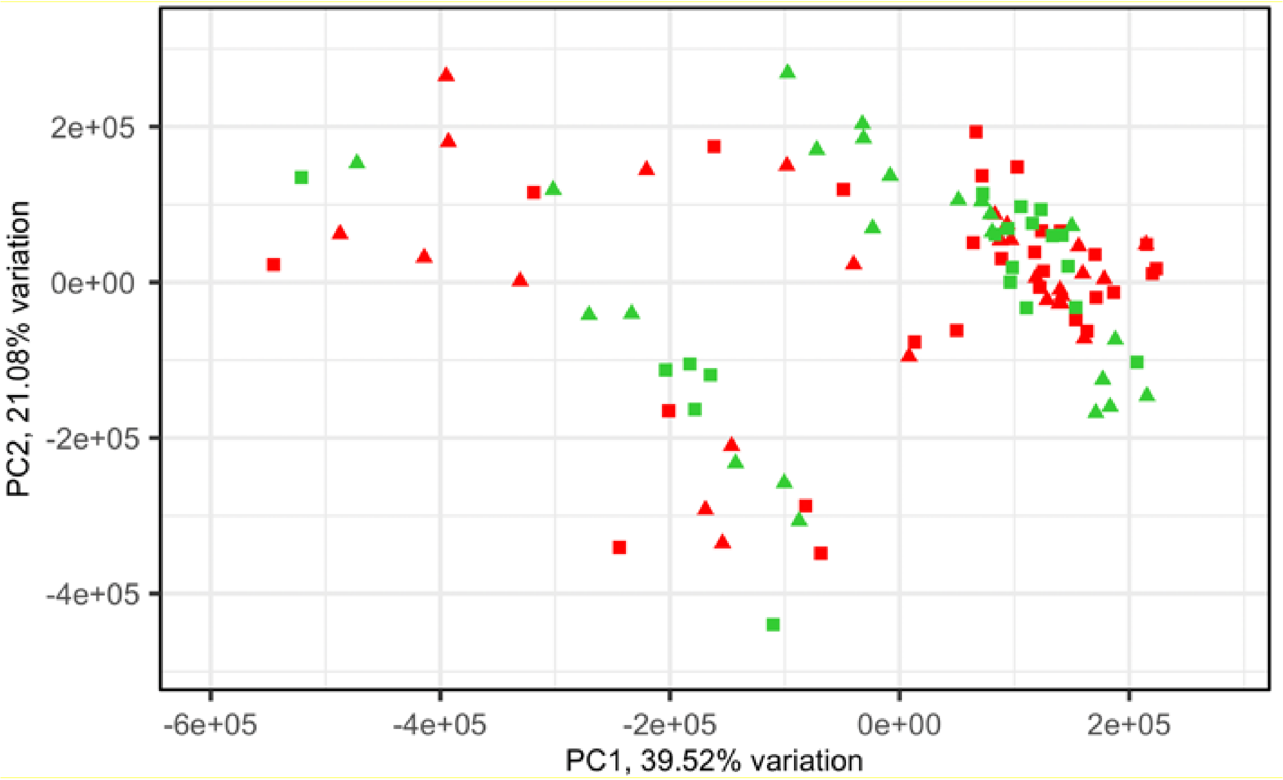

© 2024 Montaldo P et al. *JAMA Network Open.*

eFigure 2. Identification of any sources of variance in the high-income countries dataset

Two-dimensional principal component analysis plot of mean centering and scaling based on the complete gene expression of HIE babies and healthy controls (HIC). The patients have different colors according to gestational age, gender, birthweight. Square shapes represent healthy controls and triangle shapes represent HIE babies. Each dot represents the expression profile of a patient at a time point. Different colors depict a different birth weight (2000, 2500, 3000, 3500, 4000), gestational age (36, 37, 38, 39, 40, 41) and gender (males, females).

© 2024 Montaldo P et al. *JAMA Network Open.*

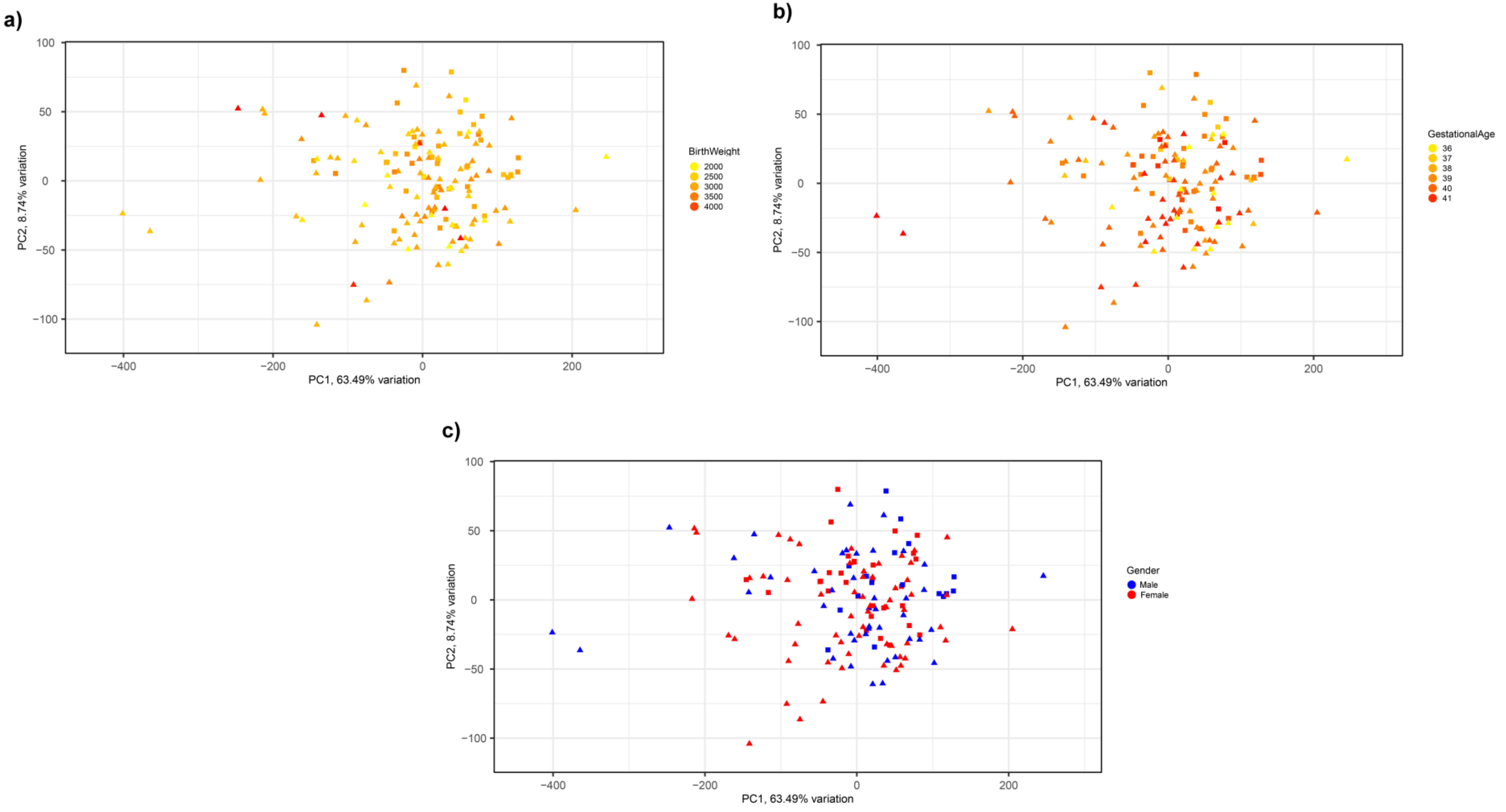

© 2024 Montaldo P et al. *JAMA Network Open.*

eFigure 3. Identification of any sources of variance in the low-and-middle-income countries dataset

Two-dimensional principal component analysis plot of mean centering and scaling based on the complete gene expression of HIE babies from batches 1 and 2 (LMIC). The patients have different colors according to gestational age, gender, birthweight. Square shapes represent good outcome and triangle shapes represent adverse outcome. Each dot represents the expression profile of a patient at T0. Different colors depict a different birth weight (2000, 2500, 3000, 3500, 4000), gestational age (36, 37, 38, 39, 40, 41) and gender (males, females).

© 2024 Montaldo P et al. *JAMA Network Open.*

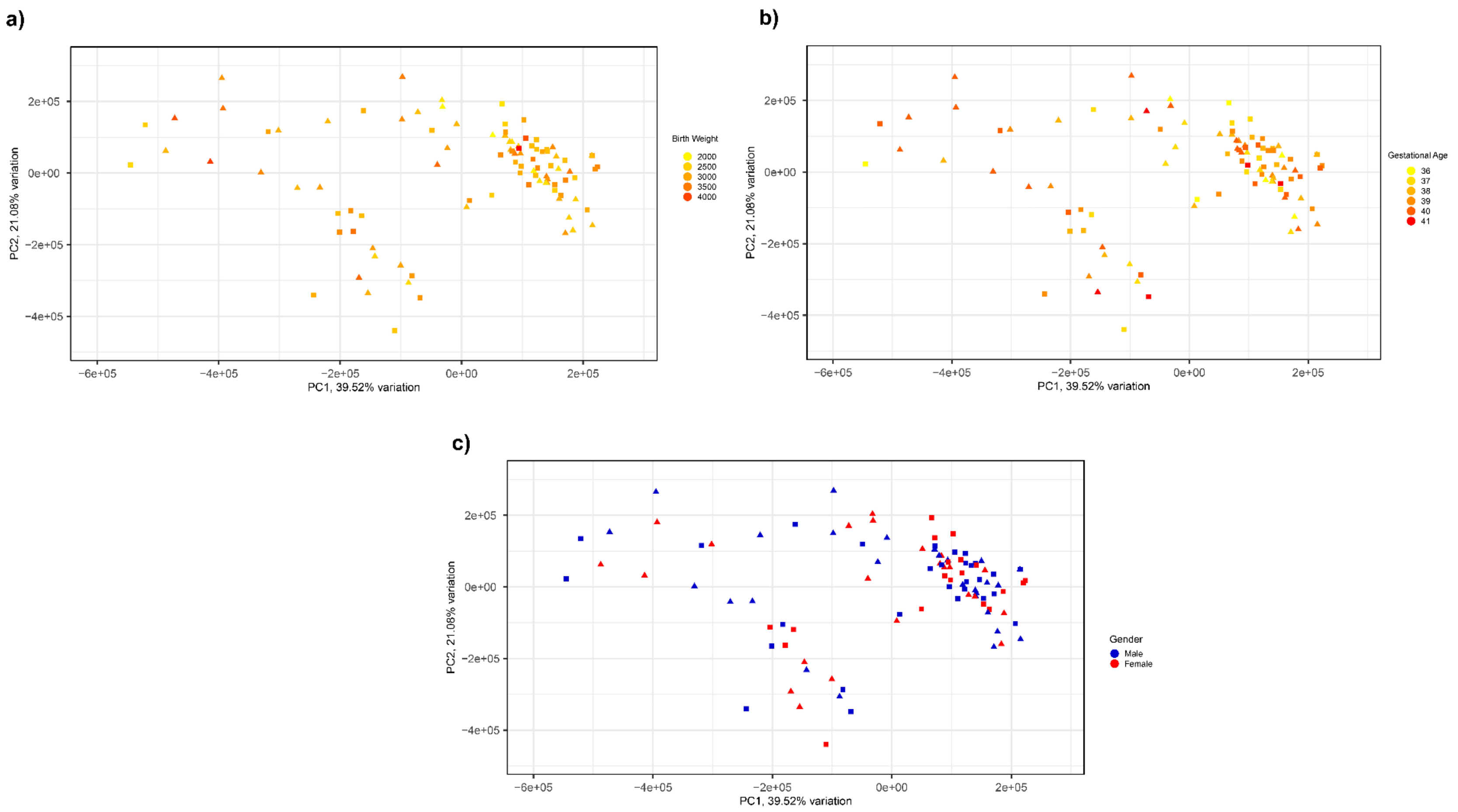

© 2024 Montaldo P et al. *JAMA Network Open.*

eFigure 4. Genome expression profile after birth in neonates with HIE with adverse outcome

Volcano plot showing the significant genes identified in the comparison of neonates with adverse versus good outcome in high income countries (**a**) and low-and-middle-income countries (**b**). The genes are plotted according to log2 fold-change (x axis) and log10 p value (y axis). In red are genes with false discovery rate (FDR) < 0.05 and fold change >1. In blue are shown those genes with only FDR < 0.05 and in green the genes with only a fold change >1.

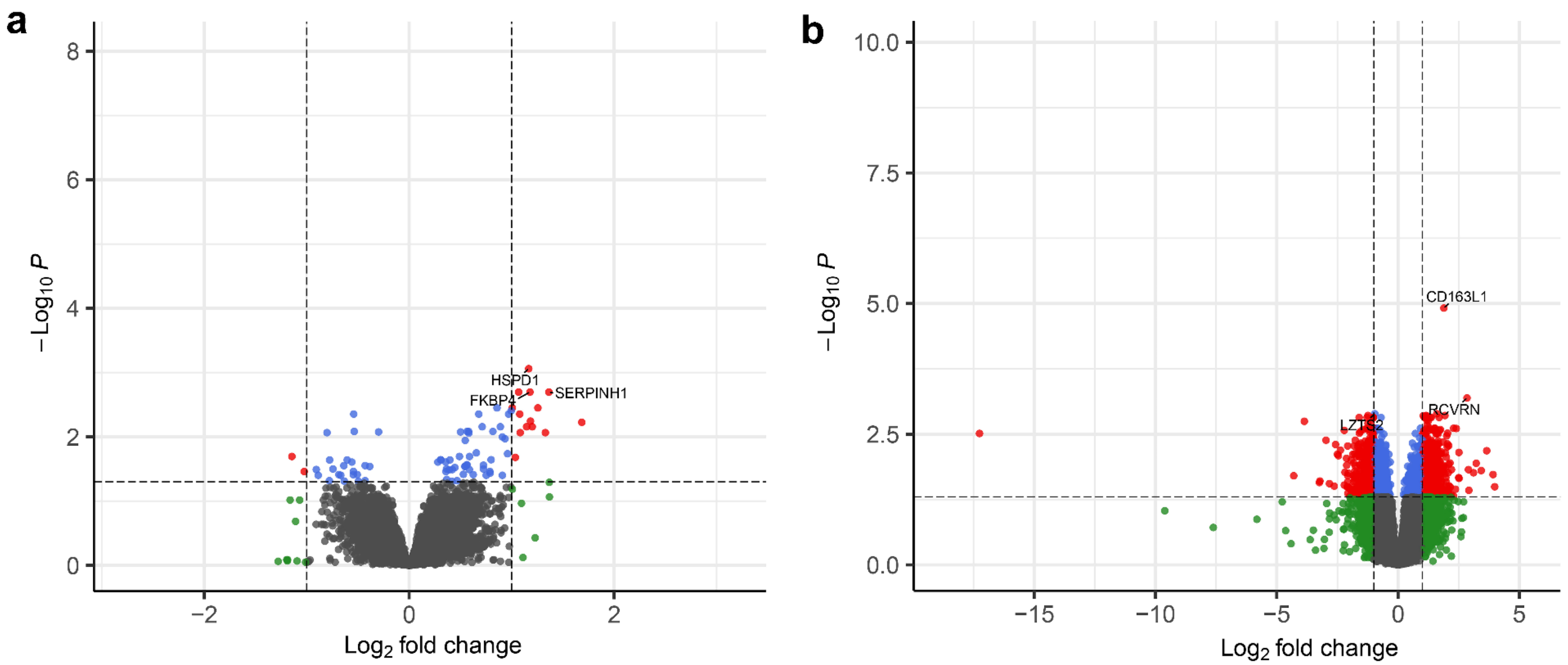

© 2024 Montaldo P et al. *JAMA Network Open.*

eFigure 5. Temporal evolution of genome expression of neonates with HIE as opposed to healthy controls

Temporal expression of 2306 significant genes in the clusters 5 to 9 after comparison of HIE neonates versus healthy controls. Time points (6=T0, 24=T1, 48=T2, 72=T3) are plotted along the X axis and gene expression values are plotted along the Y axis. Solid lines have been drawn to show the actual average value of gene expression at each time point. Fitted curves are displayed as dotted lines.

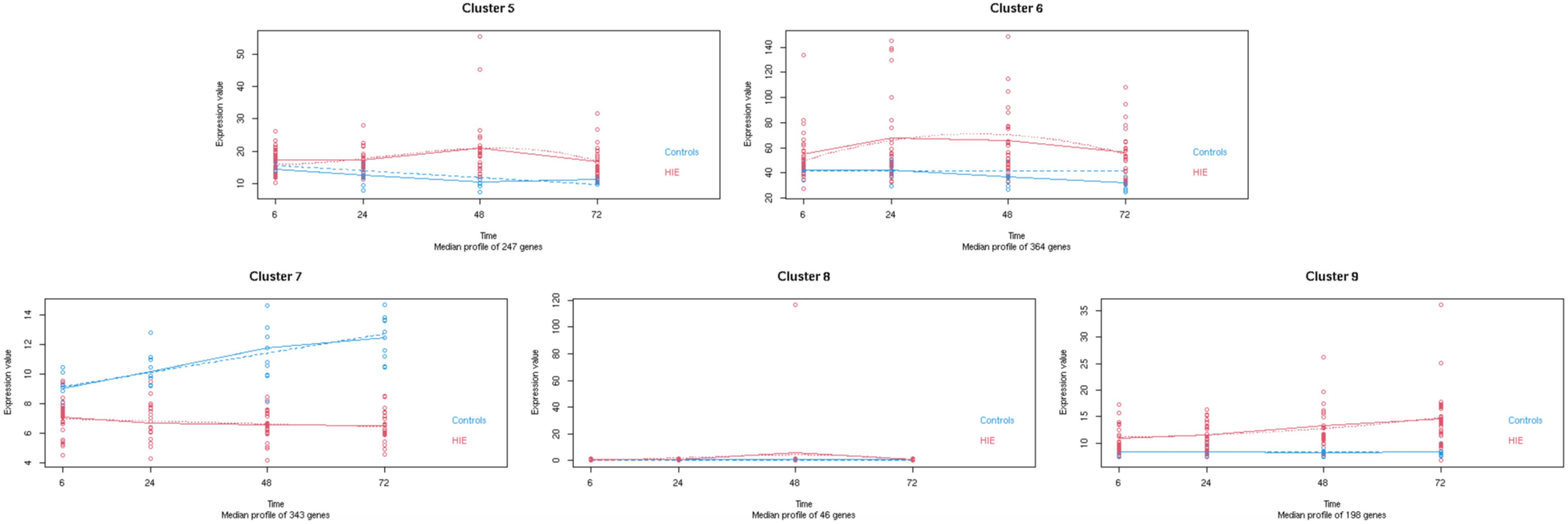

© 2024 Montaldo P et al. *JAMA Network Open.*
