# Supplementary material for: Whole-Blood Gene Expression Profile After Hypoxic-Ischemic Encephalopathy
Source: JAMA Netw Open. 2024 Feb 2;7(2):e2354433. doi: 10.1001/jamanetworkopen.2023.54433 (PMC10837749; doi:10.1001/jamanetworkopen.2023.54433)
Supplement: Supplement 3. — Data Sharing Statement [file jamanetwopen-e2354433-s003.pdf]

Data Sharing Statement

Montaldo. Whole-Blood Gene Expression Profile After Hypoxic-Ischemic Encephalopathy. *JAMA Netw Open*. Published February 02, 2024. doi:10.1001/jamanetworkopen.2023.54433

Data
Data available: No

Additional Information
Explanation for why data not available: Data used the study can be accessed from the published supplementary files
